# Betulinic Acid Enema Alleviates Colitis by Targeting Vitamin D Receptor to Protect the Intestinal Mucosal Barrier and Modulate Mucosa‐Associated Microbiota

**DOI:** 10.1002/ptr.70404

**Published:** 2026-06-18

**Authors:** Lina Dong, Yuanxia Gao, Qian Li, Guangxin Chen, Dong Yang, Liping Zhao, Xiaojing Liu, Shumei Lin

**Affiliations:** ^1^ Core Laboratory, Shanxi Provincial People's Hospital Affiliated to Shanxi Medical University Taiyuan Shanxi People's Republic of China; ^2^ Department of Infectious Diseases The First Affiliated Hospital of Xi'an Jiaotong University Xi'an Shaanxi People's Republic of China; ^3^ Institutes of Biomedical Sciences, Shanxi University Taiyuan Shanxi People's Republic of China; ^4^ Clinical Laboratory, Shanxi Provincial People's Hospital Affiliated to Shanxi Medical University Taiyuan Shanxi People's Republic of China; ^5^ Department of Pathology Shanxi Provincial People's Hospital Affiliated to Shanxi Medical University Taiyuan Shanxi People's Republic of China

**Keywords:** betulinic acid, intestinal barrier, microbiota, ulcerative colitis, VDR

## Abstract

Ulcerative colitis (UC) is a chronic inflammatory disease of the colon with limited effectiveness and safety of current drug therapies. Betulinic acid (BA) is a naturally occurring pentacyclic triterpenoid present in traditional Chinese medicines used clinically as retention enemas for UC, but its direct effects and mechanisms remain unclear. This study evaluated the therapeutic impact of BA enema in dextran sulfate sodium (DSS)‐induced colitis by monitoring disease activity index (DAI), colon length, and histopathology, and by assessing inflammatory cytokines, mucosal tight junction, and mucin‐2 (MUC2) expression. Colonic mucosa‐associated microbiota were profiled by 16S rRNA gene sequencing. Potential BA targets were predicted using Swiss Target Prediction and validated by molecular docking, molecular dynamics simulations, cellular thermal shift assay, and biolayer interferometry. The functional relevance of the vitamin D receptor (VDR) was examined using a VDR antagonist in vivo and in vitro, and transcriptomic datasets from UC patients were analyzed to define VDR expression patterns. BA enema markedly attenuated DSS‐induced colitis, such as reducing the DAI, inhibiting colonic damage, and improving colon length shortening. The BA also significantly restored the tight junction proteins, associated mucin proteins, and mucin‐2 (MUC2). The BA reduced potentially pathogenic bacteria in the colonic mucosa. In addition, BA significantly restored the VDR proteins, and the VDR antagonist weakened the effects of BA in vivo and in vitro. The binding site between VDR and BA was Trp286. Transcriptomic analyses confirmed VDR downregulation in UC. These findings indicate that BA enema alleviates experimental colitis in part by targeting VDR to protect the intestinal mucosal barrier and modulate mucosa‐associated microbiota, supporting BA as a promising candidate for UC treatment.

**Abbreviations**
BA: betulinic acid
BLI: biological layer interference
CETSA: cellular thermal shift assay
DAI: disease activity index
DSS: dextran sulfate sodium
H&E: hematoxylin and eosin
PAS: periodic acid Schiff
TCM: traditional Chinese medicine
UC: ulcerative colitis
VDR: vitamin D receptor

## 1 Introduction

Ulcerative colitis (UC) is a chronic inflammatory bowel disease characterized by relapsing inflammation that primarily affects the colon and rectum. UC is associated with substantial morbidity, frequent relapse, therapeutic refractoriness in a subset of patients, and an increased risk of colorectal cancer, classifying it as a refractory disease (Li et al. 2019; Nadeem et al. 2020). Its global incidence and prevalence have risen markedly in recent decades (Le Berre et al. 2023). Despite advances in medical management, current therapies remain insufficient in terms of durable efficacy and safety for many patients, highlighting the need for additional treatment options.

Enema therapy is a long‐standing and practical approach for distal colonic inflammation and is widely used in traditional Chinese medicine (TCM) (Yan et al. 2020). Compared with oral administration, rectal delivery can achieve high local exposure at inflamed mucosa with reduced systemic burden, potentially improving tolerability.

Our previous work demonstrated that aqueous retention enema of *Anemone chinensis Bunge* (Baitouweng in Chinese) markedly alleviated colitis by restoring intestinal barrier proteins and regulating the gut microbiota (Dong et al. 2022). Interestingly, as the main component in Anemone chinensis Bunge, the betulinic acid (BA) plays functional effects on UC (Dong et al. 2022). Further study demonstrated that BA was identified as a shared and potentially important active component in multiple traditional compound prescriptions clinically applied for UC (Huang et al. 2023). BA, a pentacyclic triterpenoid, displayed various biological activities, including antiviral, anti‐inflammatory, and anticancer effects (Lou et al. 2021). However, as a single compound in *Anemone chinensis Bunge*, the therapeutic efficacy of BA on UC has not been systematically elucidated.

The intestinal microbiota comprises luminal and mucosa‐associated communities, with the latter being in closer proximity to the epithelium and more strongly associated with UC activity and clinical progression than fecal microbiota (Dong et al. 2017; Nishihara et al. 2021). Studies have reported differences in mucosa‐associated microbial composition between inflamed and non‐inflamed regions in UC (Hirano et al. 2018; Walker et al. 2011), and mucosa‐associated alterations may be evident earlier than fecal changes, coinciding with symptom onset and barrier impairment (Wu et al. 2020). These observations further support a mechanistic link between mucosal dysbiosis, epithelial barrier dysfunction, and inflammatory amplification.

Accordingly, this study investigated the therapeutic effects of BA enemas in a DSS‐induced colitis model and explored potential mechanisms. We assessed disease severity, epithelial barrier integrity, and mucosa‐associated microbiota. We further integrated in silico target prediction and molecular docking with biolayer interferometry and cellular thermal shift assays to identify a direct molecular target of BA. Finally, public UC transcriptomic datasets and VDR antagonism in vivo and in vitro were used to evaluate the clinical relevance and functional contribution of the identified target. The present study aimed to investigate the protective effects of BA against experimental colitis and to define its mechanistic basis, supporting the development of BA as a novel therapeutic candidate for UC.

## 2 Materials and Methods

### 2.1 Materials and Reagents

BA (purity > 98%, Catalog number: HY‐10529) and the vitamin D receptor (VDR) antagonist MeTC7 (Catalog number: HY‐147337) were purchased from MedChemExpress (Wuhan, China). Dextran sulfate sodium (DSS, MW 40,000 Da) was obtained from Seebio (Shanghai, China, Catalog number: DCJ1606A). 5‐Aminosalicylic acid (5‐ASA, purity > 99%, catalog number: A129982) was obtained from aladdin (Beijing, China). The macrophage cell line RAW 264.7 and the intestinal epithelial cell line Caco‐2 were purchased from Haixing Bioscience (Suzhou, China), and both cell lines were used at passages 4–10. High‐glucose DMEM, fetal bovine serum (FBS), trypsin–EDTA, and penicillin–streptomycin (100 U/mL penicillin G and 100 μg/mL streptomycin) were purchased from Gibco (NY, USA). Primary antibodies ZO‐1 (catalog number: PB9234, lot number:ZP1007BP07), occludin (catalog number: A01246‐4, lot number:24XC2212I13), E‐cadherin (catalog number: PB9561 lot number:ZP2239BP39) and VDR (catalog number: PB9456, lot number:ZP1929B29), Fluoro 594 Conjugated AffiniPure Goat Anti‐Rabbit IgG (H + L) (catalog number: BA1142, lot number:BST19I23C19K42), Fluoro 488 conjugated AffiniPure Goat Anti‐Rabbit IgG (H + L) (catalog number: BA1127, lot number:BST19G02A19K27) and HRP Conjugated AffiniPure Goat Anti‐rabbit IgG (H + L) (catalog number: BA1054, lot number:BST18K21C19J54) were procured from Wuhan Boster Biological Technology Co. Ltd. (Wuhan, China). Claudin‐1 (catalog number: 2874‐1‐AP, Lot number: 00205444) and β‐actin (catalog number: AC038, lot number: 3600000833) were procuredfrom Abclonal ABclonal Technology Co. Ltd. (Wuhan, China). MUC2 (catalog number: GB11344, lot number: AC230821055) were procured from Wuhan Servicebio Technology Co. Ltd. (Wuhan, China). The rabbit IHC kit (PV‐9001) was obtained from ZSGB‐Bio (Beijing, China). The TaKaRa RNA PCR Kit (AMV) and SYBR Green Master Mix were used for qPCR. ELISA Kit was obtained from Shanghai Jianglai Biotechnology Co. Ltd. (Shanghai, China). The periodic acid‐Schiff (PAS) staining regent was obtained from Solarbio (Beijing, China). Unless otherwise specified, all other reagents were of analytical grade.

### 2.2 Animal Studies

Female C57BL/6J mice (6–8 weeks old, weight 18–22 g) were obtained from the animal center of Shanxi provincial people's hospital and housed under specific pathogen‐free conditions (12 h light/dark cycle, 20°C–25°C, 50% ± 10% humidity) with ad libitum access to chow and water. Animal studies adhered to hospital guidelines and received approval from the Shanxi provincial people's hospital animal ethics committee (no. 2021‐05, no. 2025‐669).

Experimental colitis was induced by administration of DSS in drinking water as described previously with minor modifications (Chen et al. 2021; Eichele and Kharbanda 2017; Jing et al. 2021). Briefly, 40 mice were randomly assigned to five groups with equal group sizes (*n* = 8 per group): (i) control group with normal drinking water; (ii) DSS group receiving 3% (w/v) DSS in drinking water for 7 days followed by PBS enema for 7 days; (iii) BA high concentration (BAH) group with 3% (w/v) DSS and 20 mg/kg BA enema for 7 days; (iv) BA low concentration (BAL) group with 3% (w/v) DSS and 10 mg/kg BA enema for 7 days; (v) 5‐ASA group with 3% (w/v) DSS and 100 mg/kg 5‐aminosalicylic acid (5‐ASA) enema for 7 days.

To facilitate further mechanistic exploration, 32 mice were randomly assigned to four groups with equal group sizes (*n* = 8 per group): (i) control group with normal drinking water; (ii) DSS group receiving 3% (w/v) DSS in drinking water for 7 days followed by PBS enema for 7 days; (iii) BA group with 3% (w/v) DSS and 20 mg/kg BA enema for 7 days; (iv) Antagonist group with 3% (w/v) DSS and both 20 mg/kg BA enema and MeTC7 administered intraperitoneally at a dose of 10 mg/kg/day for 7 days.

All drug treatments were administered after DSS exposure, indicating a curative treatment strategy rather than a preventive intervention. In designing and reporting this study, we took into account recent best‐practice recommendations for pharmacological research on natural products/phytopharmacology, including guidance on experimental design, material characterization, and transparent reporting (Heinrich et al. 2019; Wang et al. 2024).

Rectal delivery was selected to maximize local exposure in the inflamed distal colon and to mimic the clinical use of retention enemas for distal UC. For enema administration, mice were anesthetized using isoflurane inhalation anesthesia (2%–3%, v/v, for induction, 1%–2%, v/v, for maintenance). BA was dissolved in a small volume of DMSO and then diluted with 0.5% carboxymethylcellulose sodium (CMC‐Na) containing 0.1% Tween‐80 to the final concentration required. The final DMSO concentration was kept below 2% (v/v). A total volume of 100 μL of BA, 5‐ASA, or PBS was slowly administered into the colon via a flexible catheter inserted 2–3 cm from the anal verge. Enemas were administered once daily for 7 consecutive days. After administration, mice were held in a vertical position for approximately 30 s to prevent leakage.

### 2.3 Colitis Model Evaluation

Animal model evaluation was performed to monitor the disease severity using the parameters disease activity index (DAI), body weight loss, stool consistency and fecal blood as described publication (Cooper et al. 1993). At the end of the experiment, mice were humanely euthanized using an approved anesthetic‐based euthanasia method. The colon length (from the ileocecal junction to the anal verge) was measured. Colon tissues were fixed in 10% (v/v) neutral buffered formalin, paraffin‐embedded, sectioned, and stained with hematoxylin and eosin (H&E). Histological scoring was performed in a blinded manner according to Chen et al. (2017).

### 2.4 Immunohistochemistry (IHC) and PAS Staining

Paraffin sections (4 μm) from colon tissues were deparaffinized and rehydrated. Antigen retrieval was performed in EDTA buffer (pH 9.0) using a pressure cooker. Endogenous peroxidase activity was quenched with 3% (v/v) H_2_O_2_, followed by overnight incubation at 4°C with primary antibodies against ZO‐1, occludin, claudin‐1, E‐cadherin, or MUC2 (1:500). Sections were processed with the PV‐9001 kit, developed using DAB, and counterstained with hematoxylin. Mucin content was evaluated by PAS staining.

### 2.5 RNA Extraction and Quantitative Real‐Time PCR (qPCR)

Total RNA was extracted from colon tissues using universal RNA mini Kit and reverse‐transcribed with the TaKaRa RNA PCR kit. The qPCR was performed using SYBR Green master mix on a Bio‐Rad real‐time PCR system. Gene expression was normalized to β‐actin and calculated using the 2^−ΔΔCT^ method. Primer sequences are provided in Table S1.

### 2.6 DNA Extraction and 16S rDNA Amplification

Considering that the BAL group showed insufficient therapeutic efficacy and that the mechanism of the positive control (5‐aminosalicylic acid) may differ from that of BA, to avoid mechanistic confounding, only the control, DSS, and BAH groups were included in the microbiota analysis. On Day 7, colonic tissues from the control, DSS, and BAH groups were collected for analysis of mucosa‐associated microbiota. Luminal contents were gently removed prior to sampling. Intestinal segments were repeatedly washed with sterile PBS. Genomic DNA was extracted using the MagPure Soil DNA LQ Kit. The 16S rRNA V3–V4 region was amplified using the following primers: forward primer 341F (5′‐CCTACGGGNGGCWGCAG‐3′) and reverse primer 806R (5′‐GGACTACHVGGGTWTCTAAT‐3′). Libraries were constructed using the KAPA Hyper Prep Kit and sequenced on the Illumina NovaSeq platform with paired‐end 2 × 250 bp reads. The average sequencing depth per sample and the total effective reads after quality control were recorded. QIIME was used for OTU clustering, representative sequence selection, and downstream analyses. OTU composition, diversity indices, and taxonomic profiles from phylum to genus were compared across groups. Multiple‐testing corrections were applied: Tukey's HSD for diversity indices and FDR (Benjamini–Hochberg) for differential abundance. Bray–Curtis distance‐based PCoA was used to evaluate between‐group differences in community structure. Group‐level differences were tested using PERMANOVA (vegan package, R). Differential taxa were identified using DESeq2 (version 1.26.0) based on raw read counts, with Wald tests and FDR correction.

### 2.7 Target Prediction of BA

Putative BA targets were predicted using SwissTargetPrediction based on BA structure. UC‐related genes were retrieved from GeneCards and filtered by relevance score. Overlapping genes between BA‐predicted targets and UC‐related genes were defined as candidate targets for mechanistic investigation.

### 2.8 Molecular Docking

Molecular docking was performed to predict the binding mode and affinity between BA and target proteins. The 3D structure of BA (PubChem CID: 64971) was retrieved from PubChem in SDF format, and target protein structures were downloaded from the Protein Data Bank (PDB) with corresponding PDB IDs. Protein structures were prepared in PyMOL (v2.1.0) by removing water molecules and co‐crystallized ligands, and then processed in AutoDock Tools (v1.5.6) to generate PDBQT files. Docking simulations were implemented in PyRx (v0.8) with AutoDock Vina (v2.0). The grid box was defined to cover the protein active site (typically 30 × 30 × 30 Å^3^, grid spacing 0.375 Å), with default parameters (exhaustiveness = 8, maximum 9 binding modes). Binding affinities (kcal/mol) were recorded, and top‐ranked poses were visualized via PyMOL (3D, non‐covalent interactions) and Discovery Studio 2020 (2D, interacting residues).

### 2.9 Bio‐Layer Interferometry (BLI)

His‐tagged proteins were immobilized on Ni–NTA biosensors, and reference sensors without protein were used for background subtraction. BA was dissolved in DMSO and diluted in PBST (PBS with 0.02%, v/v, Tween‐20); PBST alone served as blank. Measurements were performed on an Octet analyzer with baseline (PBST, 60 s), association (BA, 120 s), and dissociation (PBST, 60 s) phases. Dual referencing (sensor and buffer) was applied. Association (Kon), dissociation (Kdis), and equilibrium (KD) constants were calculated.

### 2.10 Cellular Thermal Shift Assay (CETSA)

To exclude potential cytotoxic effects of BA, cell viability was evaluated prior to CETSA. Raw 264.7 and Caco‐2 cells were treated with increasing concentrations of BA (0–50 μM) for the indicated time period, and cell viability was assessed using a cell viability assay. No significant cytotoxicity was observed at concentrations of 20 μM, indicating that the BA concentration used in CETSA and subsequent experiments was non‐cytotoxic.

CETSA was performed in both RAW 264.7 macrophages and Caco‐2 cells. Cells were lysed in RIPA buffer containing protease inhibitors, sonicated, and centrifuged to remove insoluble debris. Equal amounts of cell lysates were incubated with BA (20 μM) or an equivalent volume of DMSO at 37°C for 1 h, followed by heating across a temperature gradient (46°C–67°C). Samples were then cooled to room temperature and centrifuged, and the soluble fractions were collected for analysis. Soluble proteins were separated by SDS‐PAGE and immunoblotted using an anti‐VDR antibody (1:1000). Band intensities were quantified using ImageJ, and thermal stability curves were generated based on the relative soluble VDR levels at each temperature point.

### 2.11 Molecular Dynamics Simulation

Docking at ligand‐binding sites defined from co‐crystallized ligands was performed using AutoDock Vina to identify key interacting residues. Alanine scanning was conducted in Schrödinger to estimate ΔΔG values and prioritize residues for further analysis. Molecular dynamics simulations (100 ns) were performed for wild‐type and mutant complexes, and trajectories were analyzed in ChimeraX. Binding free energies (Δ*G*) were estimated using MM‐PBSA to assess the impact of mutations on ligand–protein interactions.

### 2.12 Public Dataset Analysis

To investigate the expression pattern of VDR in UC, three publicly available human transcriptomic datasets (GSE87466, GSE92415, and GSE165512) were retrieved from GEO. Dataset characteristics were summarized as follows: GSE87466: mucosal biopsies from adult UC patients with moderately to severely active disease (UC *n* = 87) and normal controls (*n* = 21). Tissue is mucosal biopsy, consistent with colonic mucosa sampling in UC studies. GSE92415: colon mucosal biopsies from UC patients with moderately to severely active disease (UC *n* = 162, including baseline and week‐6 samples in PURSUIT‐SC induction study) and healthy controls (*n* = 21). GSE165512: biopsies from IBD patients, including UC (*n* = 40), Crohn's disease (*n* = 84), and controls (*n* = 46). Tissue is biopsy‐based, but colonic mucosa is not explicitly stated in the GEO summary.

All subsequent data preprocessing, normalization, integration, and statistical analyses were performed in the R statistical environment (version 4.1.3). Raw expression data were log_2_(x + 1) transformed and normalized using normalizeBetweenArrays (limma). The three datasets were merged into a single expression matrix; batch effects were corrected using sva. The effectiveness of batch correction was evaluated by (1) boxplots of expression distributions before/after correction and (2) PCA, showing clustering by biological condition rather than dataset origin. These QC plots are provided in Figure S1.

Following data integration and quality control, the expression values of the VDR gene were extracted from the combined matrix. Differential expression analysis comparing UC tissues and non‐inflamed control tissues was performed using the linear modeling framework implemented in the limma package. Empirical Bayes moderation was applied to standard errors to improve statistical power, and *p* values were adjusted for multiple testing using the Benjamini–Hochberg false discovery rate (FDR) method.

The results of differential expression analysis—including log_2_ fold‐changes (logFC), adjusted *p* values (adj.*p*.val), and significance status—were visualized using the ggplot2 package, typically via volcano plots, boxplots, or violin plots to illustrate VDR expression levels and statistical differences between UC and control groups.

### 2.13 VDR Immunofluorescence Staining of Colonic Tissues

Colon sections were deparaffinized, rehydrated, permeabilized with 0.1% (v/v) Triton X‐100, and blocked. Samples were incubated with anti‐VDR antibody followed by fluorophore‐conjugated secondary antibody, and images were acquired by fluorescence microscopy.

### 2.14 Effects of BA on Cell Viability

Effects of BA on various cell viabilities were assessed by CCK‐8. RAW264.7 macrophages or caco2 or co‐culture (1:1) were seeded in 96‐well plates, then treated with various concentrations of BA for 24 h. One hundred microliters of 10% CCK‐8 (DOJINDO, Japan) solution was added to each well. Then, the absorbance was measured at 450 nm using a microplate reader. Based on the CCK8 assay, the treatment concentrations of each test sample which did not exhibit cytotoxicity, were determined (data not shown).

### 2.15 Co‐Culture and TEER Measurement

RAW 264.7 and Caco‐2 cells were maintained in high‐glucose DMEM supplemented with 10% FBS and 1% penicillin–streptomycin, respectively. For co‐culture, Caco‐2 cells (1.0 × 10^5^/well) were seeded on Transwell inserts (0.4 μm, Corning) and cultured for ~20 days with medium replacement every 2–3 days until stable TEER was achieved. RAW 264.7 cells (2.0 × 10^5^/well) were seeded in the lower chamber 2 days before treatment. Co‐cultures were assigned to four groups: Control, LPS (5 μg/mL), LPS + BA (5 μg/mL LPS + 20 μM BA), and LPS + BA + MeTC7 (5 μg/mL LPS + 20 μM BA +500 nM MeTC7). After 24 h, epithelial barrier integrity was assessed using a cell impedance analyzer. Experiments were repeated three times. TEER was measured using an EVOM to assess the intestinal epithelial barrier integrity.

### 2.16 Co‐Culture, ELISA, Immunofluorescence Staining, and Western Blot Analysis

RAW 264.7 and Caco‐2 cells were co‐cultured in Transwell plates with glass coverslips. After Caco‐2 monolayers were fully established (~20 days), RAW 264.7 cells were added and treated as described in Section 2.15 for 24 h.

Four ELISA cell culture supernatants were collected after treatment and centrifuged at 1000 × g for 10 min to remove debris. The concentrations of TNF‐α, IL‐6, and IL‐1β were quantified using commercially available ELISA kits according to the manufacturer's instructions. Briefly, standards and samples were added to pre‐coated 96‐well plates and incubated at room temperature for the recommended time. After washing, biotin‐conjugated detection antibodies and HRP‐streptavidin were added sequentially. The reaction was developed with substrate. Absorbance was measured at 450 nm using a microplate reader. Cytokine concentrations were calculated from standard curves.

For immunofluorescence staining, cells were washed with cold PBS, fixed with 4% paraformaldehyde and blocked with 10% normal goat serum. Primary antibodies against occludin, Claudin‐1, E‐cadherin, and ZO‐1 (1:200) were incubated overnight at 4°C, followed by incubation with fluorophore‐conjugated secondary antibodies and DAPI counterstaining. Images were acquired using a laser‐scanning confocal microscope under identical acquisition settings. Fluorescence signals from immunolabeled specimens were quantitatively assessed using Image‐Pro Plus 6.0 software. All captured images were split into independent single‐color channels, with parameters manually calibrated via standard built‐in modes to precisely identify fluorescent signals. Subsequently, the Count/Size module was applied to calculate the integrated optical density (IOD) values of all visual fields (Lee et al. 2024).

For Western blot analysis, Caco‐2 cells were lysed in RIPA buffer supplemented with protease inhibitors. Protein concentrations were determined using a BCA assay. Equal amounts of protein were separated by SDS‐PAGE using 7.5% gels for ZO‐1, E‐cadherin; 10% gels for occludin and 12.5% gels for Claudin‐1, and subsequently transferred onto PVDF membranes. Membranes were blocked with 5% non‐fat milk and incubated overnight at 4°C with primary antibodies against ZO‐1, E‐cadherin, occludin, and Claudin‐1 (1:1000 dilution), followed by HRP‐conjugated secondary antibodies. Protein bands were visualized using an enhanced chemiluminescence (ECL) detection system and quantified by densitometric analysis. β‐actin was used as the internal loading control. Band intensities were quantified using ImageJ.

### 2.17 Statistical Analysis

All quantitative data are presented as mean ± SEM. Statistical analyses were performed using GraphPad Prism 8.0 (CA, USA). Data normality was assessed prior to hypothesis testing. For comparisons among multiple groups, one‐way analysis of variance (ANOVA) followed by Tukey–Kramer HSD post hoc test was applied. A two‐tailed *p* < 0.05 was considered statistically significant.

For gene expression analysis, qPCR data were normalized to the internal control gene β‐actin and calculated using the 2^−ΔΔCt^ method. Public transcriptomic datasets were log_2_‐transformed and normalized using the limma package in R, as described in Section 2.12.

## 3 Results

### 3.1 Betulinic Acid Enemas Attenuate DSS‐Induced Colitis in Mice

In animal experiment, it was observed that the DSS induced progressive body weight loss (Days 4–7), increased DAI, loose stools, and hematochezia compared with the control (Figure 1A–C). The body weight or DAI did not change in BAL group, whereas the body weight in BAH and 5‐ASA group significantly increases (*p* < 0.001 vs. DSS) and DAI significantly decreases (*p* < 0.001 vs. DSS). DSS also caused colonic congestion and shortening (*p* < 0.0001 vs. control), which was alleviated by BAH and 5‐ASA (*p* < 0.01 vs. DSS) but not BAL (*p* = 0.8795) (Figure 1D,E). Histology showed severe mucosal injury in DSS mice and improvement with BAH or 5‐ASA, with reduced histological scores (*p* < 0.01 vs. DSS), BAL had no apparent effect (Figure 1F,G). For cytokine expression, the DSS treatment resulted to upregulation of IL‐1b, TNF‐α, IL‐6, COX‐2 and iNOS, the cytokine expression significantly decreases in BAH and 5‐ASA group, which was not observed in BAL group (Figure 1H–L).

**FIGURE 1:**
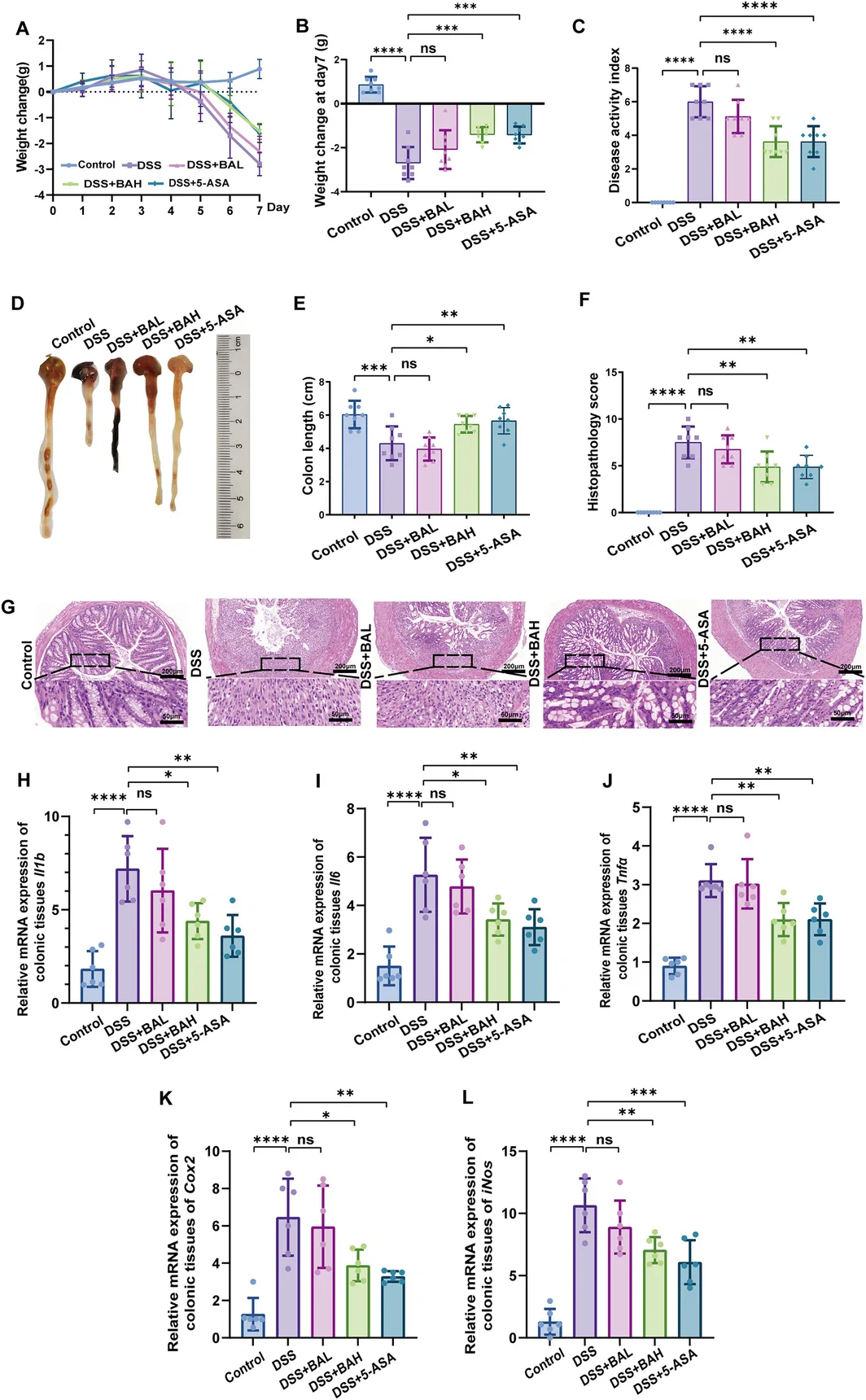
Betulinic acid (BA) attenuates DSS‐induced colitis in mice (*n* = 8 per group). (A) Body weight changes during the experimental period. (B) Quantification of body weight loss at Day 7. (C) Disease Activity Index (DAI) scores. (D) Representative images of colons from each experimental group at Day 7. (E) Quantitative analysis of colon length. (F) Histopathological scores evaluating inflammation severity and tissue injury. (G) Representative hematoxylin and eosin (H&E)‐stained colon sections showing histopathological alterations (scale bar: 200 μm). The boxed areas indicate regions shown at higher magnification. (H–L) Quantitative real‐time PCR (qPCR) analysis of pro‐inflammatory mediators in colonic tissues: (H) Il‐1b, (I) Il‐6, (J) Tnf‐α, (K) Cox2, and (L) iNos mRNA expression levels. Data are presented as mean ± SEM. Statistical analyses were performed using one‐way ANOVA followed by Tukey–Kramer HSD post hoc test. *p < 0.05, **p < 0.01, ***p < 0.001, ****p < 0.0001; ns, not significant.

### 3.2 BA Restores Tight Junction Proteins and the Mucus Layer in DSS‐Induced Colitis

Figure 2 showed that the intensity of occludin, ZO‐1, E‐cadherin, and claudin‐1 expression in colonic epithelium treated by DSS was lower than control, indicating tight junction disruption in epithelium. In BAH and 5‐ASA groups, the intensity of protein expression was increased than the DSS group, suggesting that tight junction protein expression was restored. It was not observed in BAL, compared to DSS. Furthermore, DSS also resulted in depleted goblet cells and reduced mucus production. PAS staining and MUC2 immunostaining showed marked mucus layer disruption. BAH and 5‐ASA preserved MUC2 and mucus layer integrity, while BAL did not (Figure 2).

**FIGURE 2:**
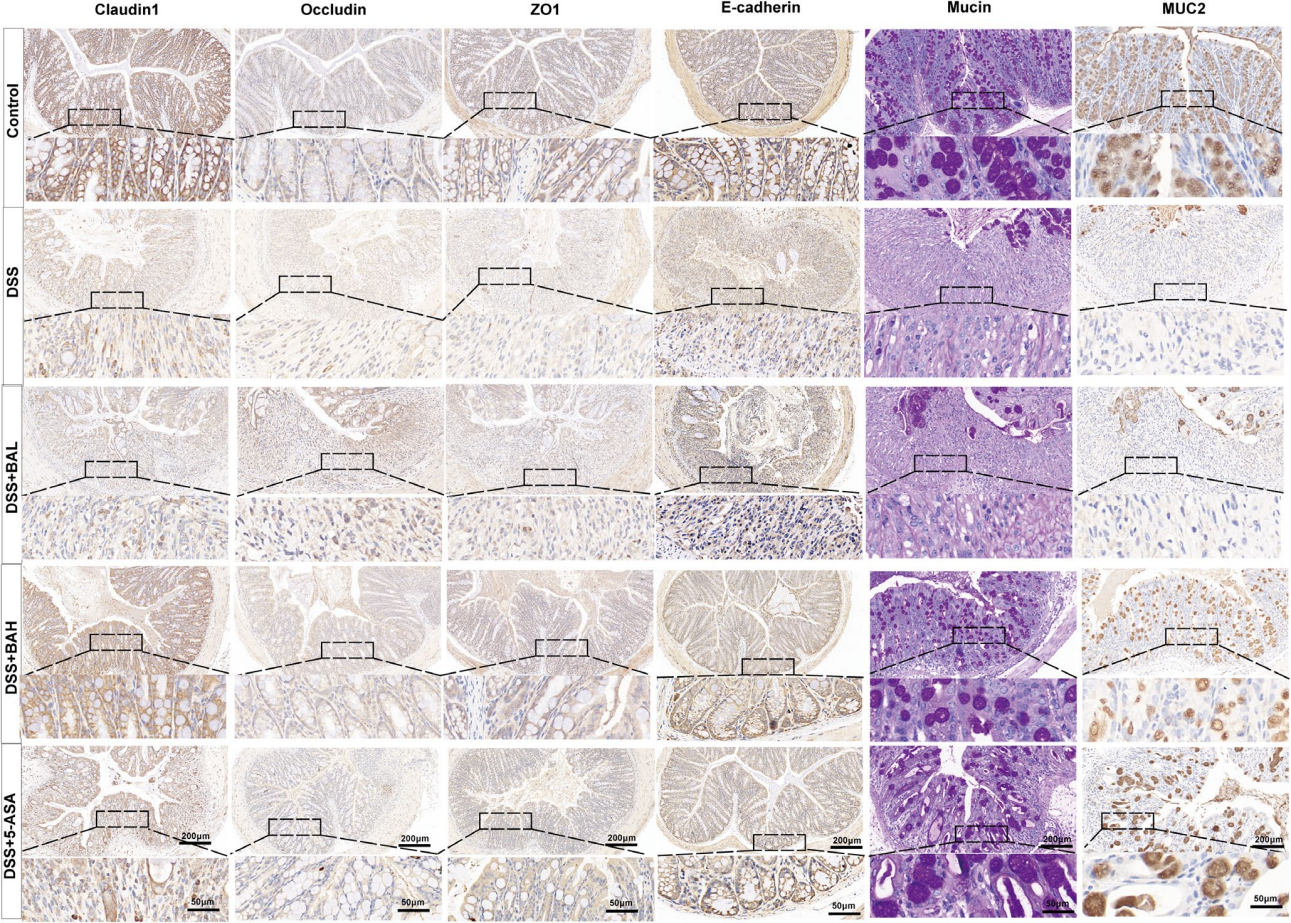
Betulinic acid (BA) restores intestinal mucosal barrier integrity (*n* = 3 per group). Representative immunohistochemical staining of the tight junction proteins claudin‐1, occludin, ZO‐1, E‐cadherin, and MUC2, along with mucin content assessed by periodic acid–Schiff (PAS) staining (scale bar: 200 μm). The boxed areas indicate regions shown at higher magnification. [Correction added on 6 August 2026, after first online publication: Figure 2 has been corrected.]

### 3.3 BA Modulates Mucosa‐Associated Microbiota in DSS‐Treated Mice

Given that BAL exhibited no in vivo efficacy, we profiled the mucosa‐associated microbiota in the control, DSS, and BAH groups (Figure 3). BAH did not significantly alter α‐diversity, as measured by the Shannon index (Figure 3A), nor did it affect phylum‐level community composition (Figure 3B). At the genus level, BAH attenuated the DSS‐associated enrichment of *Enterococcus*, *Escherichia*–*Shigella*, and *Turicibacter* (Figure 3C–E), while inducing only minimal changes in most other genera.

**FIGURE 3:**
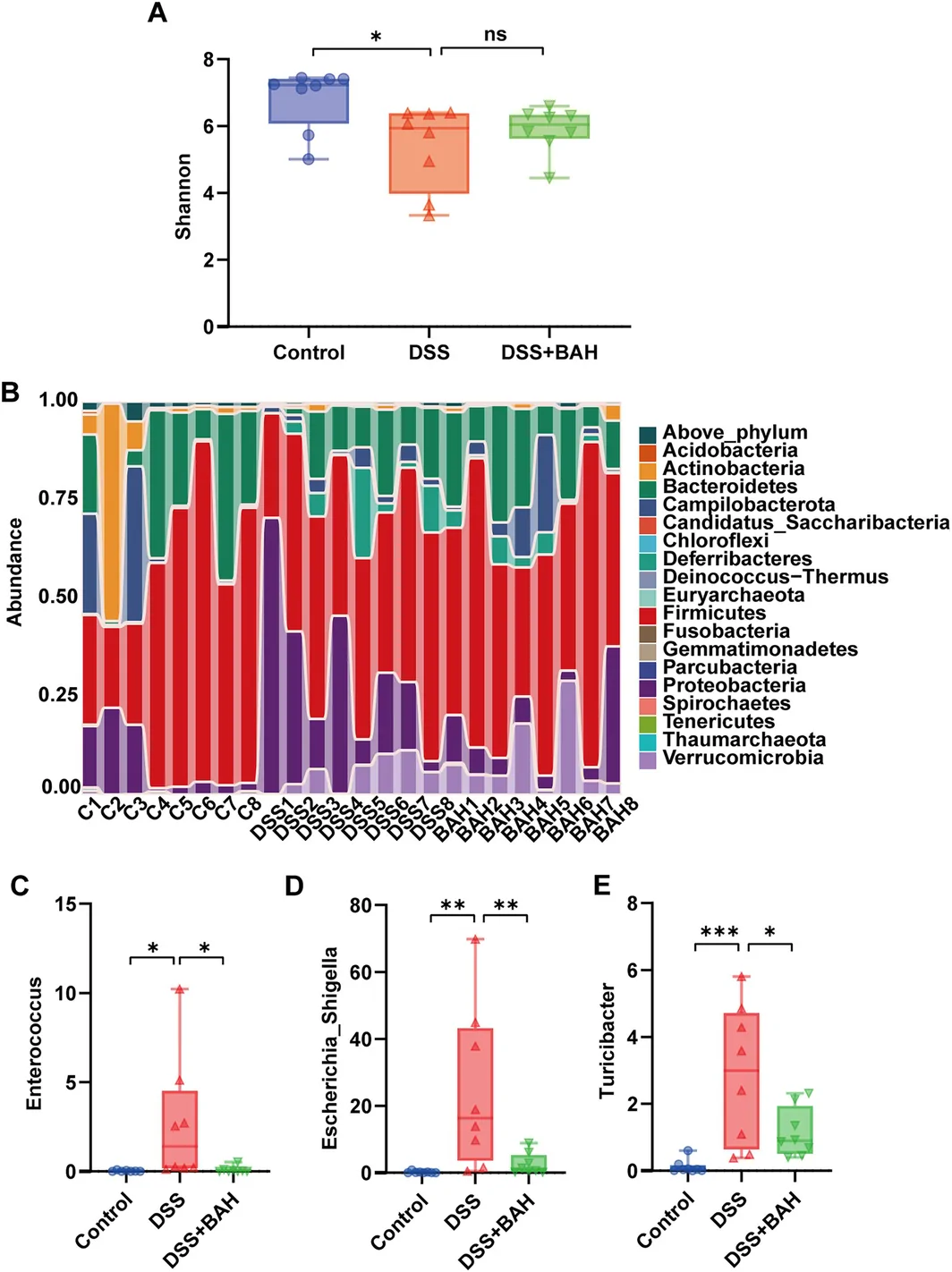
Betulinic acid (BA) modulates the composition of mucosa‐associated microbiota (*n* = 8 per group). Mucosa‐associated microbiota from colonic tissues were analyzed by 16S rRNA gene sequencing (*n* = 8 per group). (A) Shannon diversity index. (B) Relative abundance of microbial taxa at the phylum level. (C–E) Relative abundance of the genera (C) *Enterococcus*, (D) *Escherichia–Shigella*, and (E) *Turicibacter*. Data are presented as mean ± SEM. Statistical analyses were performed using one‐way ANOVA followed by Tukey–Kramer HSD post hoc test. *p < 0.05, **p < 0.01, ***p < 0.001, ns, not significant.

### 3.4 Target Prediction and Validation Identify VDR as a BA‐Binding Protein

SwissTargetPrediction identified 54 putative targets of BA. After intersecting these targets with UC‐related genes, 10 candidate targets were obtained (Figure 4A,B). Molecular docking analysis ranked VDR as the top predicted binding partner with a binding energy of −14.1 kcal/mol (Figure 4C). Combined with relevance scores from GeneCards (Figure 4D), COX‐2, PPARG, and VDR were selected for validation.

**FIGURE 4:**
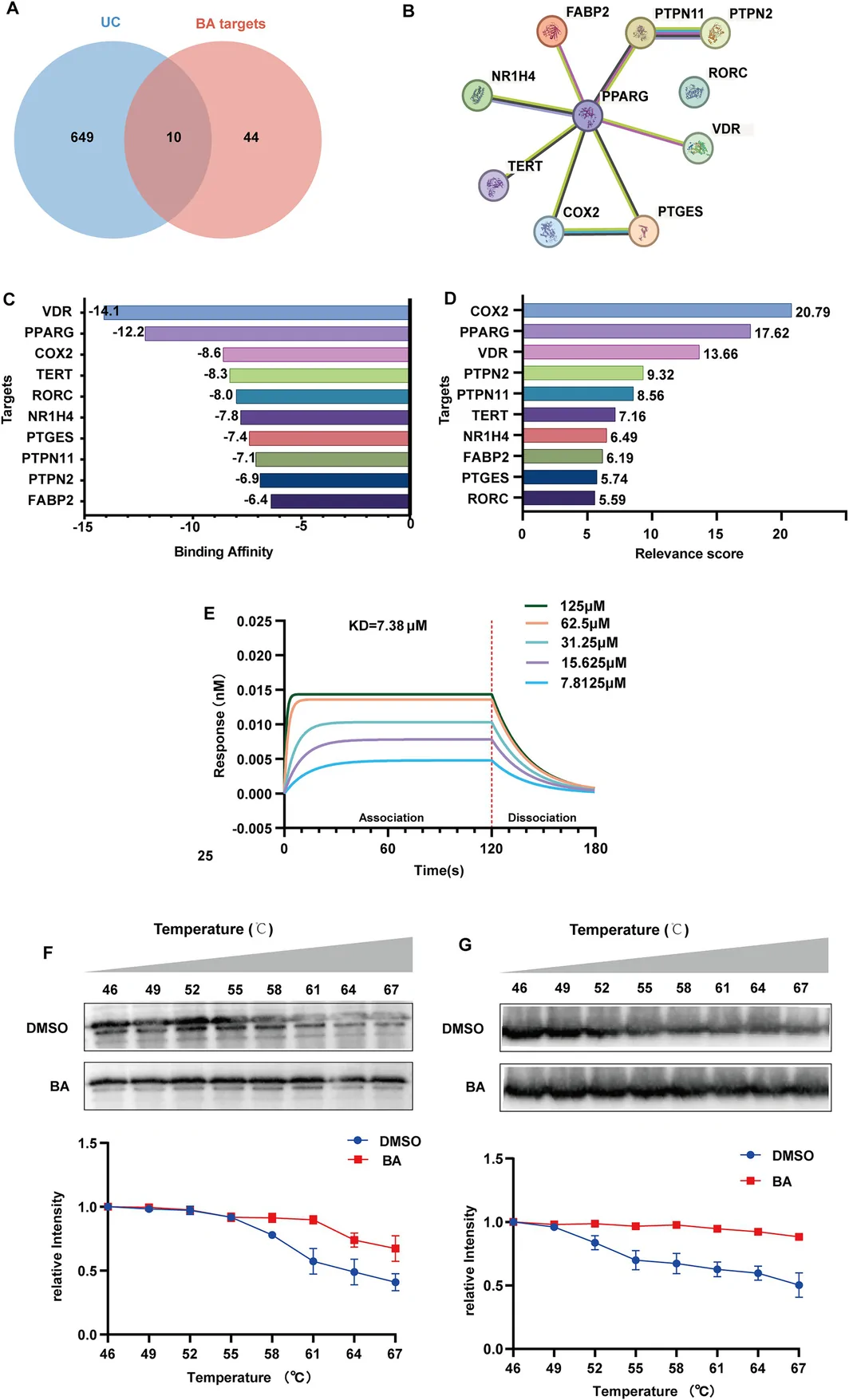
Betulinic acid (BA) directly targets the vitamin D receptor (VDR). (A) Venn diagram illustrating the overlap between BA‐predicted targets and ulcerative colitis (UC)–related genes. (B) Protein–protein interaction (PPI) network of the overlapping targets constructed using the STRING database. (C) Molecular docking analysis showing the binding energies of BA with the predicted targets. (D) Relevance scores of the overlapping targets in ulcerative colitis (UC). (E) Bio‐layer interferometry (BLI) analysis depicting the binding kinetics and affinity of BA to VDR (Kd value). (F) Cellular thermal shift assay (CETSA) demonstrating BA‐induced thermal stabilization of the VDR protein of raw 264.7 (*n* = 3). (G) CETSA demonstrating BA‐induced thermal stabilization of the VDR protein of caco‐2 (*n* = 3).

Bio‐layer interferometry (BLI) assay confirmed a direct binding interaction between BA and VDR (Figure 4E), whereas no binding was detected between BA and COX‐2 or PPARG. Specifically, VDR bound to BA with moderate affinity, characterized by a KD of 7.38 μM, a Kon of 6.67 × 10^3^ (1/Ms), a Kdis of 4.99 × 10^−2^ (1/s), and a fitting curve *R* ^2^ of 0.6757.

To exclude potential cytotoxic effects of BA, cell viability was assessed using a CCK‐8 assay. Treatment with BA at concentrations up to 50 μM did not significantly affect the viability of either RAW 264.7 macrophages or Caco‐2 cells, indicating that the concentrations used in subsequent experiments were non‐cytotoxic. CETSA further supported a direct interaction between BA and VDR. Compared with the DMSO control, BA treatment increased the amount of soluble VDR remaining after heating across the indicated temperature range, resulting in a higher relative VDR signal at elevated temperatures (Figure 4F,G). These results indicate that BA stabilizes VDR against thermal denaturation, consistent with BA binding to VDR in cells.

Molecular docking simulations revealed that BA binds VDR primarily through hydrogen bonding (with residues Try143 and His305) and extensive hydrophobic interactions (e.g., with TRP286, TYR295, and adjacent aliphatic residues) (Figure 5A). During 100 ns molecular dynamics simulations, residues Ser278, Trp286, His305, Ser306, and His397 exhibited high interaction fractions (> 0.5) with BA (Figure 5B). Notably, the hydroxyl/carboxyl groups of BA formed frequent direct or water‐mediated hydrogen bonds with Ser278, His305, Ser306, and His397, while hydrophobic interactions with Trp286 and Tyr295 contributed to the stabilization of the BA‐VDR complex (Figure 5C). Alanine scanning mutagenesis identified Trp286 as the most critical residue for BA binding, as its mutation resulted in the largest ΔΔG value (Figure 5D). Mutation of Trp286 also increased the root mean square deviation (RMSD) and overall structural fluctuation of the BA‐VDR complex (Figure 5E). Subsequent structural analysis and root mean square fluctuation (RMSF) analysis further confirmed that the Trp286‐centered pocket (encompassing His305 and Tyr295) plays a key role in mediating BA binding to VDR (Figure 5F–I).

**FIGURE 5:**
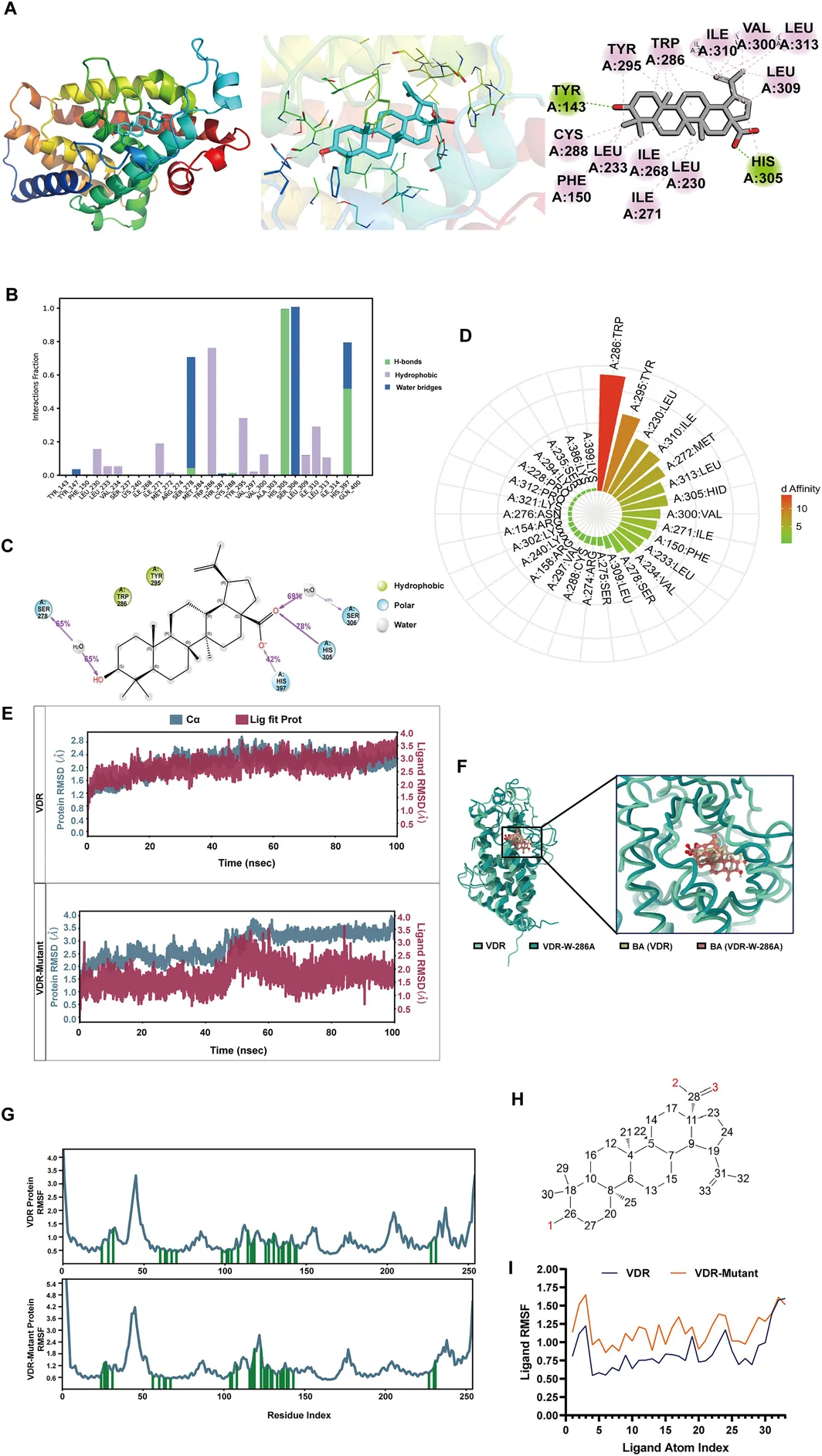
Molecular basis of the BA–VDR interaction. (A) Molecular docking pose of betulinic acid (BA) within the VDR ligand‐binding pocket. (B) Per‐residue contribution analysis of key amino acids at the BA–VDR binding interface. (C) Two‐dimensional interaction map of the BA–VDR binding mode derived from molecular dynamics (MD) simulations. (D) Alanine‐scanning mutagenesis of VDR showing predicted binding energy changes (Δaffinity) for the top 30 residues. (E) Root‐mean‐square deviation (RMSD) profiles of VDR (wild‐type) and the Trp286Ala mutant during MD simulations. (F) Structural comparison of VDR conformations between wild‐type and Trp286Ala mutant, highlighting differences in the ligand‐binding pocket. (G) Root‐mean‐square fluctuation (RMSF) analysis of VDR (wild‐type vs. mutant); green markers indicate key residues involved in BA binding. (H) Atomic numbering scheme of BA. (I) RMSF profiles of BA when complexed with wild‐type or mutant VDR.

### 3.5 VDR Is Downregulated in UC Datasets and Preserved by BA in DSS Colitis

In merged UC transcriptomic datasets, VDR mRNA was significantly reduced in UC samples compared with controls (*p* < 0.0001; Figure 6B), consistent with global differential expression (Figure 6A,B). In DSS colitis, epithelial VDR signal was reduced and partially preserved by BA at a high concentration (Figure 6C,D).

**FIGURE 6:**
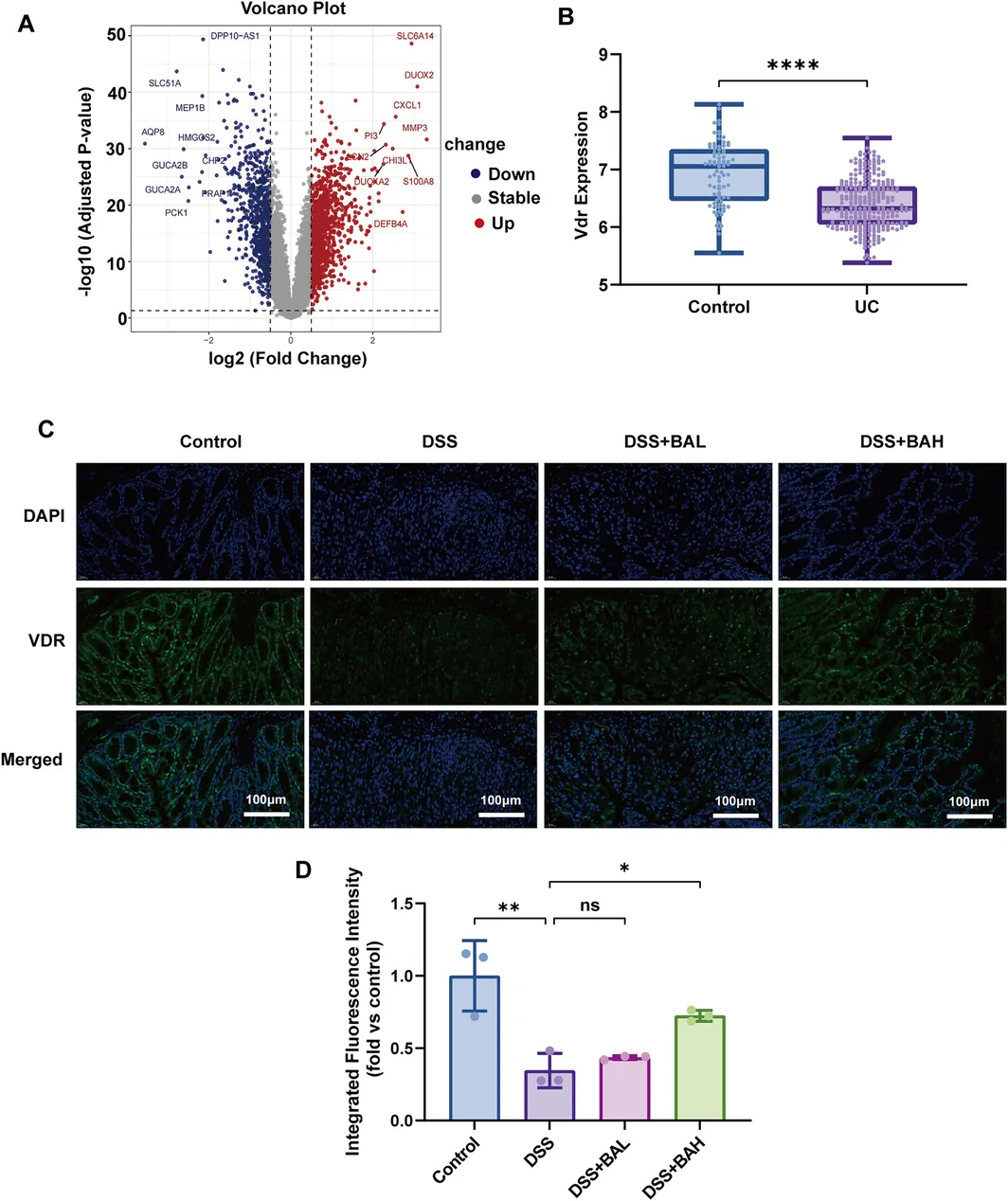
Public data analysis reveals VDR dysregulation in ulcerative colitis (UC) and immunofluorescence staining of VDR in mouse colon tissues. (A) Volcano plot showing differentially expressed genes (DEGs) between control and UC samples, with significantly upregulated and downregulated genes highlighted. (B) Boxplot comparing VDR expression levels between control and UC groups in public transcriptomic datasets. Statistical significance was assessed using Student's *t*‐test; ****p < 0.0001. (C) Representative immunofluorescence staining of VDR in colon tissues from control, DSS, DSS + BAL, and DSS + BAH mice. Nuclei were counterstained with DAPI (blue), and VDR is shown in green. Scale bar = 100 μm. (D) Quantitative analysis of the average fluorescence intensity. Data are presented as mean ± SEM. Statistical comparisons were performed using one‐way ANOVA followed by Tukey–Kramer HSD post hoc test. Statistical significance is indicated as *p < 0.05, **p < 0.01; ns, not significant (vs. DSS group) (*n* = 3 per group).

### 3.6 VDR Antagonism Abolishes BA‐Mediated Protection In Vivo

MeTC7 largely abrogated BA‐mediated improvements in DSS colitis, including body weight loss (Figure 7A,B), DAI (Figure 7C), colon shortening (Figure 7D,E), histology and mucosal injury (Figure 7F,G).

**FIGURE 7:**
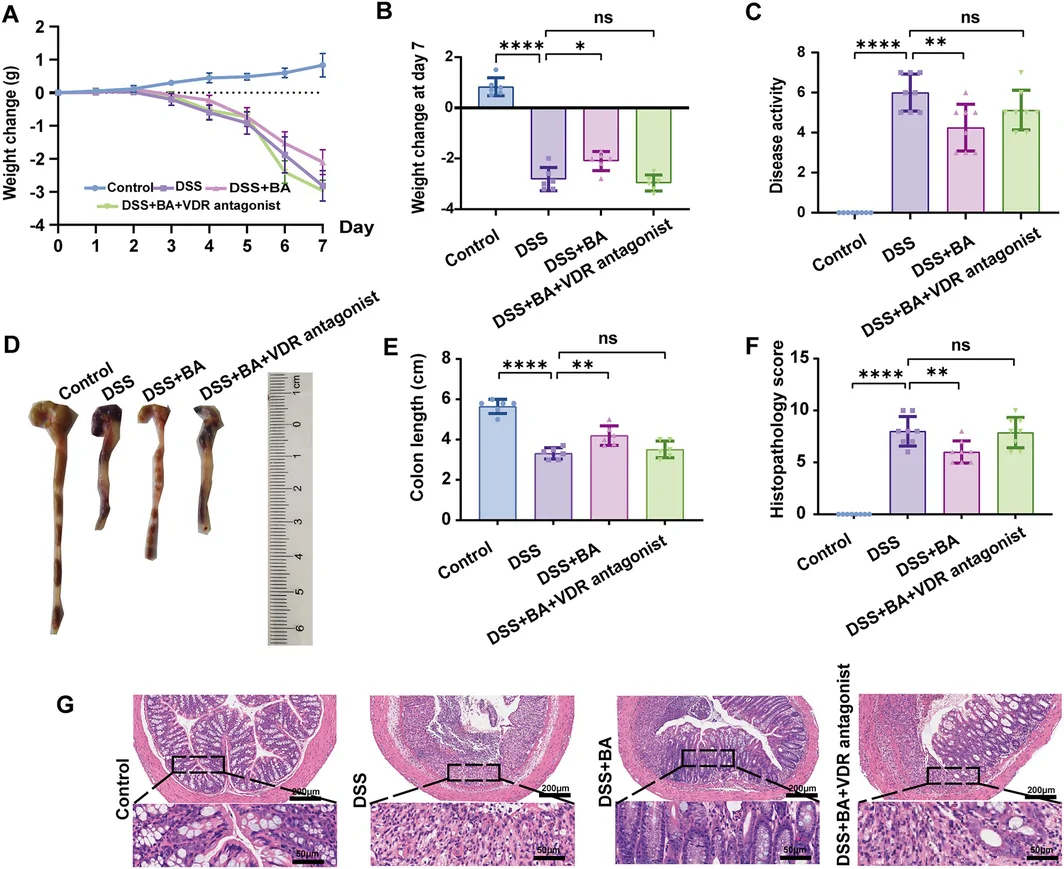
The VDR antagonist MeTC7 reverses the protective effects of betulinic acid (BA) in vivo (*n* = 8 per group). (A) Body weight changes during the experimental period. (B) Quantification of body weight loss at Day 7. (C) Disease Activity Index (DAI) scores. (D) Representative images of colons from each experimental group at Day 7. (E) Quantitative analysis of colon length. (F) Histopathological scores evaluating inflammation severity and tissue injury. (G) Representative hematoxylin and eosin (H&E)‐stained colon sections showing histopathological alterations (scale bar: 200 μm). The boxed areas indicate regions shown at higher magnification. Data are presented as mean ± SEM. Statistical comparisons were performed using one‐way ANOVA followed by Tukey–Kramer HSD post hoc test. Statistical significance is indicated as *p < 0.05, **p < 0.01, ****p < 0.0001; ns, not significant (vs. DSS group).

### 3.7 VDR Antagonism Abolishes BA‐Mediated Protection In Vitro

Using a Transwell‐based RAW 264.7/Caco‐2 co‐culture model (Figure 8A), LPS challenge markedly impaired epithelial barrier integrity, as indicated by a significant decrease in TEER signals (Figure 8B), accompanied by increased cytokine concentration of IL‐1β, TNF‐α, IL‐6 (Figure 8C), and decreased tight junction protein expression in Caco‐2 cells, including ZO‐1, occludin, E‐cadherin, and claudin‐1 (Figure 9). BA treatment substantially restored barrier function and reversed the LPS‐induced disruption of tight junction proteins. Notably, co‐treatment with the VDR antagonist MeTC7 largely abolished BA‐mediated recovery of TEER and attenuated the restoration of tight junction proteins, indicating that BA protects epithelial barrier integrity in a VDR‐dependent manner. Consistently, immunofluorescence staining and Western blot further confirmed that BA preserved the junctional distribution of ZO‐1/occludin/Claudin‐1/E‐cadherin in Caco‐2 monolayers under LPS stimulation, whereas MeTC7 weakened these protective effects.

**FIGURE 8:**
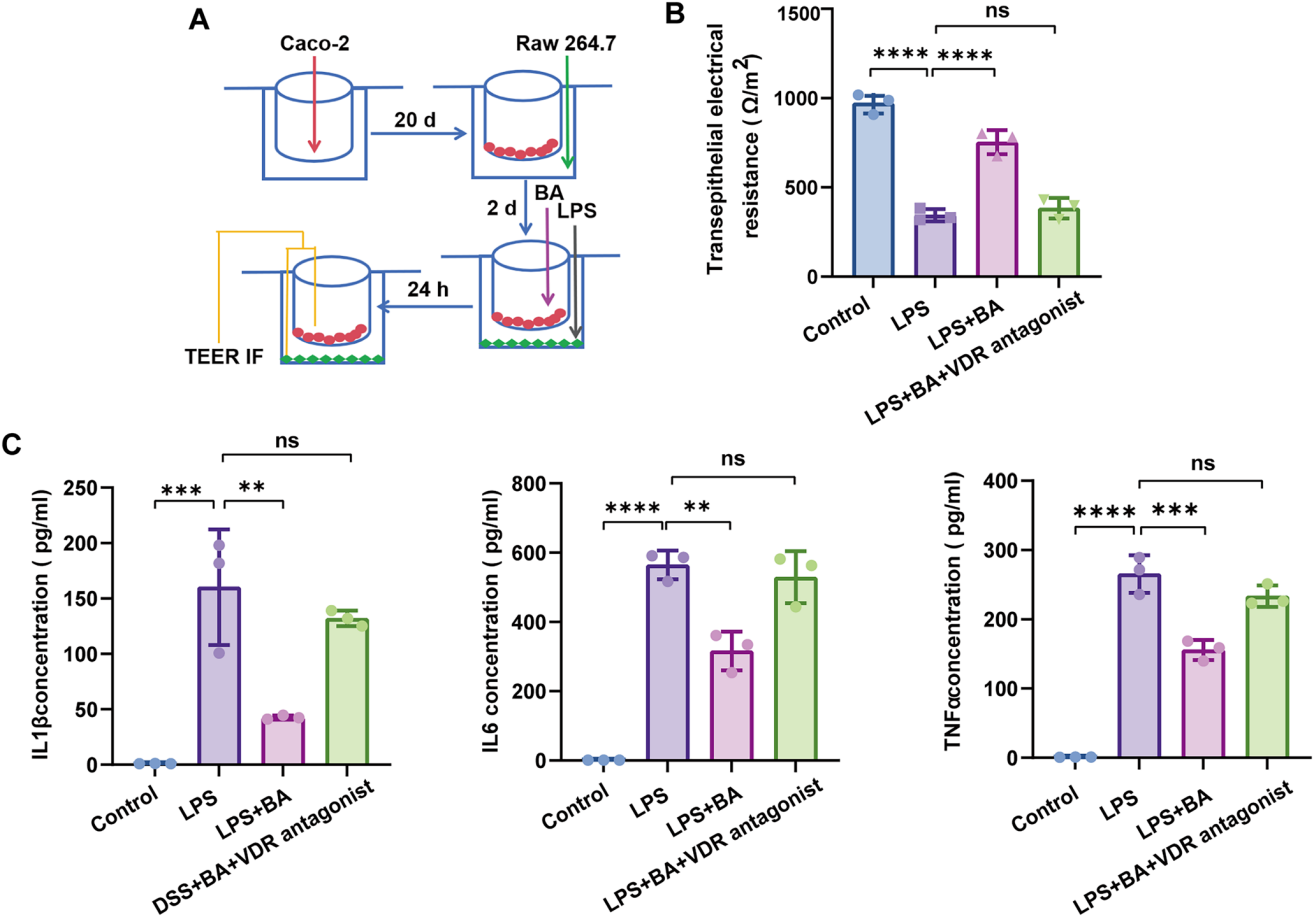
The VDR antagonist MeTC7 attenuates the protective effects of betulinic acid (BA) on intestinal barrier function in vitro (*n* = 3). (A) Schematic illustration of the in vitro co‐culture model. (B) Quantification of TEER values, showing BA‐mediated restoration of epithelial barrier integrity following LPS‐induced injury and its attenuation by VDR antagonist. (C) The concentrations of TNF‐α, IL‐6, and IL‐1β. Data are presented as mean ± SEM. Statistical comparisons were performed using one‐way ANOVA followed by Tukey–Kramer HSD post hoc test. Statistical significance is indicated as **p < 0.01, ***p < 0.001, ****p < 0.0001; ns, not significant (vs. LPS group).

**FIGURE 9:**
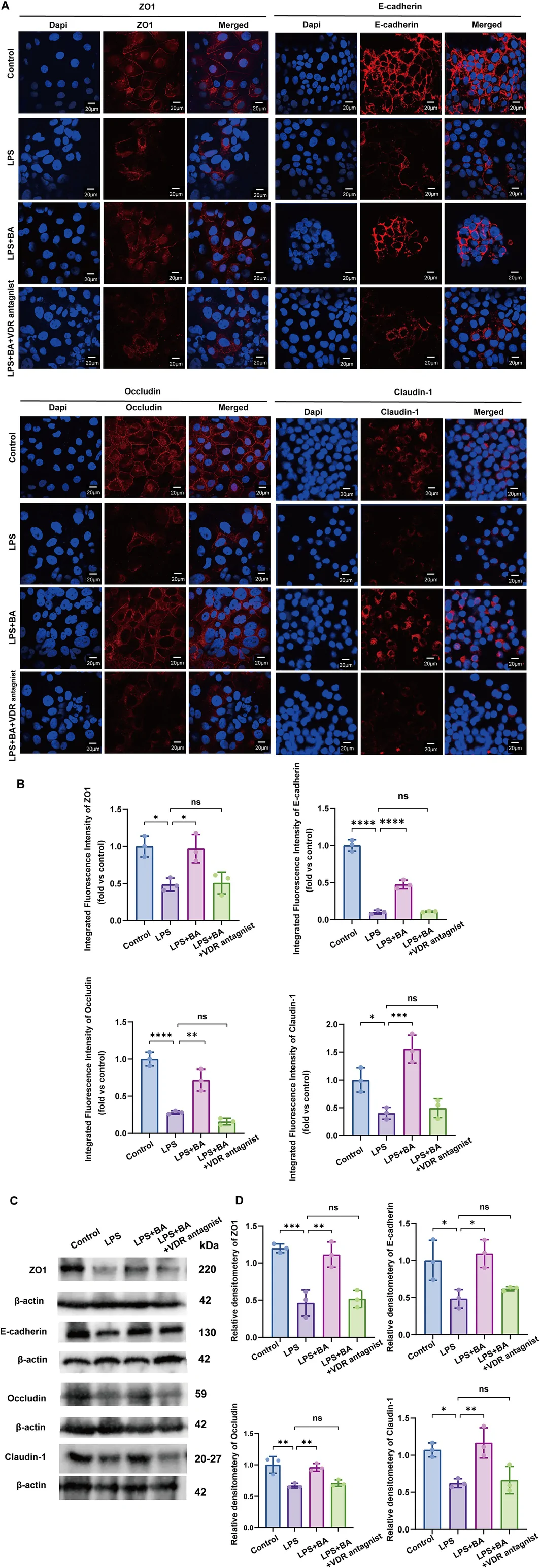
The VDR antagonist MeTC7 attenuates the protective effects of betulinic acid (BA) on intestinal barrier protein in vitro (*n* = 3). (A) Representative immunofluorescence images of the tight junction proteins ZO‐1, E‐cadherin, occludin, and claudin‐1 in Caco‐2 cell monolayers under the indicated treatments. Scale bar = 20 μm. (B) Quantitative analysis of the average fluorescence intensity. (C) Representative Western blot images of ZO‐1, E‐cadherin, occludin, and claudin‐1 protein expression. (D) Quantitative analysis of protein expression levels based on densitometry. Data are expressed as mean ± SD. Statistical comparisons were performed using one‐way ANOVA followed by Tukey–Kramer HSD post hoc test. Statistical significance is indicated as *p < 0.05, **p < 0.01, ***p < 0.001, ****p < 0.0001.

## 4 Discussion

This study evaluated the efficacy and underlying mechanism of BA administered via enema in a DSS‐induced colitis model. We demonstrated that BA exerts a protective effect in vivo, as evidenced by improved clinical indices and histopathological features, preservation of epithelial barrier components, changes in mucosa‐associated microbiota, and direct binding to the VDR–functionally required for BA‐mediated protection.

Epithelial barrier disruption is a hallmark of UC and facilitates luminal antigen translocation to drive sustained inflammation (Sandle and Rajendran 2025). In our DSS‐induced model, high‐dose BA alleviated disease activity and tissue injury, while restoring the expression of key tight junction proteins (ZO‐1, occludin, E‐cadherin and claudin‐1) and preserving goblet cell/mucus barrier markers (PAS and MUC2). These findings indicate that BA stabilizes epithelial barrier integrity at both the tight junction and mucus layers, thereby limiting continuous mucosal stimulation and downstream inflammatory response. Consistent with this, BA also downregulated colonic expression of pro‐inflammatory cytokines (TNF‐α, IL‐6, and IL‐1β) and inflammatory mediators (COX‐2 and iNOS), which are established drivers of mucosal injury in UC. While these anti‐inflammatory effects may reflect direct immunomodulatory activity of BA, they are also consistent with secondary attenuation of inflammation following barrier preservation (Neurath 2019). Collectively, BA disrupts the inflammatory cascade by inhibiting these key pro‐inflammatory mediators. Histological analysis (H&E staining) confirmed reduced inflammation and tissue destruction in BA‐treated mice, which correlated with enhanced mucosal healing—a critical therapeutic target in UC management (Tan et al. 2019).

High‐dose BA did not substantially alter α‐diversity or phylum‐level composition but mitigated DSS‐associated enrichment of pathogenic taxa, including *Enterococcus*, *Turicibacter*, and *Escherichia–Shigella*. Expansion of *Escherichia–Shigella* at the mucosal interface is closely linked to epithelial damage and pro‐inflammatory signaling (Chen et al. 2014), and reduction of this genus has also been reported with 5‐ASA (Cevallos et al. 2021). Notably, increased Enterococcus abundance is frequently observed under conditions of barrier disruption and intestinal inflammation, and members of this genus can behave as opportunistic pathobionts in colitis contexts. Dai et al. (2021) reported that the abundances of Escherichia–Shigella and Enterococcus were positively correlated with IL‐17 and IL‐23 levels in patients with UC, suggesting that crosstalk between the gut microbiota and the IL‐23/IL‐17 axis may contribute to UC pathogenesis. Consistently, a study observed a positive association between Escherichia–Shigella abundance and circulating IL‐6 levels (Jialing et al. 2020), a pivotal pro‐inflammatory cytokine implicated in UC disease activity. Beyond cytokine‐related links, Wang et al. (2022) further reported that Escherichia, Shigella, and Enterococcus were positively correlated with the autophagy signaling cascade, indicating a potential connection between these taxa and host autophagy regulatory pathways. Importantly, mechanistic evidence supports a causal contribution of Enterococcus to intestinal inflammation. Seishima et al. (2019) demonstrated that UC‐derived *Enterococcus faecium* (a key species within the genus Enterococcus) harbors a pro‐inflammatory genotype and can induce coliti, while Fan et al. (2021) showed that *Enterococcus faecalis* —another Enterococcus species—exacerbates experimental colitis via glucosamine metabolism. Turicibacter has likewise been reported to shift in inflammatory and barrier‐stressed states, although its role appears context‐dependent across models. Our findings thus suggest that BA may partially converge with established UC therapies in restricting pathobionts, while avoiding the broad microbiota disruption typically associated with antibiotic use. Whether BA shapes these taxa directly (e.g., via luminal exposure) or indirectly through improved barrier function remains to be elucidated.

A key mechanistic insight from this work is the identification of VDR as a direct binding target of BA. Integrating target prediction, molecular docking, and biophysical assays, we observed specific binding between BA and VDR under our experimental conditions, further validated by CETSA‐based stabilization of VDR upon BA treatment. In silico analyses confirmed a stable BA–VDR complex and highlighted Trp286 (alongside His305 and nearby residues) as critical for maintaining binding stability. Notably, Trp286 is located within the VDR β1 domain, a key region for ligand binding and a well‐characterized core site monitoring VDR functionality (Rochel et al. 2000).

These results establish a link between the BA‐VDR molecular interaction and barrier‐protective phenotypes in colitis. The clinical relevance of this axis is supported by transcriptomic data showing reduced VDR expression in UC tissues. Consistent with these datasets, DSS‐induced colitis diminished epithelial VDR signaling, and BA treatment partially preserved VDR expression in vivo. Previous studies also demonstrate an inverse correlation between colonic epithelial VDR expression levels and UC disease activity (Triantos et al. 2022). Functionally, VDR antagonism with MeTC7 largely abrogated the protective BA‐mediated benefits across disease indices and histological parameters. In a macrophage–epithelial co‐culture model, BA restored LPS‐induced barrier dysfunction (impedance) and restored tight junction protein expression, whereas VDR antagonism significantly attenuated these beneficial effects.

Several limitations in the present study should be acknowledged. The temporal relationships among VDR modulation, epithelial barrier repair, and associated microbiota alterations were not explicitly defined. Future studies incorporating time‐resolved analyses and microbiota‐transfer experiments will help to clarify whether microbial shifts act as drivers or consequences of barrier restoration, thereby refining mechanistic interpretation. Another limitation of the present study is that we were unable to determine which specific cell type primarily mediates the effects of VDR. As VDR may exert distinct functions in different cell populations, the cell‐specific role of VDR in this process remains to be clarified. Future studies using conditional VDR knockout mice will be needed to identify the key cellular source responsible for the observed effects.

## 5 Conclusion

In summary, BA enemas alleviate experimental colitis and preserve epithelial barrier function. Mechanistically, BA directly binds VDR, and intact VDR signaling is required for BA‐mediated barrier protection in vivo and in vitro. Consistent with the barrier‐protective phenotype, microbiota profiling indicates a limited, genus‐level association of BA with mucosa‐associated communities, characterized by attenuation of DSS‐associated enrichment of several inflammation‐linked taxa. These findings support VDR‐centered strategies and provide a mechanistic rationale for further development of BA‐based rectal therapy for UC.

## Author Contributions

**Qian Li:** methodology, formal analysis, investigation. **Guangxin Chen:** investigation, conceptualization. **Xiaojing Liu:** investigation, validation. **Shumei Lin:** writing – review and editing, supervision, validation, funding acquisition, conceptualization, project administration. **Liping Zhao:** investigation. **Yuanxia Gao:** methodology, formal analysis, investigation. **Lina Dong:** conceptualization, funding acquisition, visualization, writing – original draft, writing – review and editing, validation, investigation, methodology, data curation, formal analysis, supervision, resources, project administration. **Dong Yang:** validation.

## Funding

This work was supported by the Shanxi Provincial Basic Research Program Project (no. 20210302124287).

## Ethics Statement

The animal experiment was strictly following NIH Guide for the Care and Use of Laboratory Animals; maximum efforts were made to minimize animal suffering and the number of animals necessary for the capture of reliable data.

## Conflicts of Interest

The authors declare no conflicts of interest.

## Supporting information


**Figure S1:** Boxplots of gene expression distributions and PCA plots before and after batch correction. (A) Boxplots before correction, clear distributional differences among the three GEO cohorts are observed; (B) boxplots after correction, medians and ranges are aligned across cohorts, indicating effective removal of batch effects. (C) PCA plots before correction, samples separate mainly by dataset of origin; (D) PCA plots after correction, samples are more intermingled, showing reduced dataset‐driven clustering and improved integration.

**Table S1:** Primers sequence information for RT‐PCR.

## Data Availability

The data that support the findings of this study are available on request from the corresponding author. The data are not publicly available due to privacy or ethical restrictions.

## References

[ptr70404-bib-0001] Cevallos, S. A. , J.‐Y. Lee , E. M. Velazquez , et al. 2021. “5‐Aminosalicylic Acid Ameliorates Colitis and Checks Dysbiotic *Escherichia coli* Expansion by Activating PPAR‐γ Signaling in the Intestinal Epithelium.” MBio 12: 10–128. 10.1128/mBio.03227-20 PMC7845635 33468700

[ptr70404-bib-0002] Chen, G. , J. Liu , L. Jiang , et al. 2017. “Galangin Reduces the Loss of Dopaminergic Neurons in an LPS‐Evoked Model of Parkinson's Disease in Rats.” International Journal of Molecular Sciences 19: 12. 10.3390/ijms19010012 PMC5795964 29267220

[ptr70404-bib-0003] Chen, G. , Z. Yang , D. Wen , et al. 2021. “Polydatin Has Anti‐Inflammatory and Antioxidant Effects in LPS‐Induced Macrophages and Improves DSS‐Induced Mice Colitis.” Immunity, Inflammation and Disease 9: 959–970. 10.1002/iid3.455 PMC8342204 34010516

[ptr70404-bib-0004] Chen, L. , W. Wang , R. Zhou , et al. 2014. “Characteristics of Fecal and Mucosa‐Associated Microbiota in Chinese Patients With Inflammatory Bowel Disease.” Medicine (Baltimore) 93: e51. 10.1097/MD.0000000000000051 PMC4602441 25121355

[ptr70404-bib-0005] Cooper, H. S. , S. N. Murthy , R. S. Shah , and D. J. Sedergran . 1993. “Clinicopathologic Study of Dextran Sulfate Sodium Experimental Murine Colitis.” Laboratory Investigation 69: 238–249. 8350599

[ptr70404-bib-0006] Dai, Z. F. , X. Y. Ma , R. L. Yang , et al. 2021. “Intestinal Flora Alterations in Patients With Ulcerative Colitis and Their Association With Inflammation.” Experimental and Therapeutic Medicine 22: 1322. 10.3892/etm.2021.10757 PMC8495592 34630676

[ptr70404-bib-0007] Dong, L. , X. Fan , Q. Li , et al. 2022. “Anemone Chinensis Bunge Aqueous Enema Alleviates Dextran Sulfate Sodium‐Induced Colitis via Inhibition of Inflammation and Regulation of the Colonic Mucosal Microbiota.” Journal of Ethnopharmacology 288: 114916. 10.1016/j.jep.2021.114916 34998930

[ptr70404-bib-0008] Dong, L.‐N. , J.‐P. Wang , P. Liu , Y.‐F. Yang , J. Feng , and Y. Han . 2017. “Faecal and Mucosal Microbiota in Patients With Functional Gastrointestinal Disorders: Correlation With Toll‐Like Receptor 2/Toll‐Like Receptor 4 Expression.” World Journal of Gastroenterology 23: 6665–6673. 10.3748/wjg.v23.i36.6665 PMC5643287 29085211

[ptr70404-bib-0009] Eichele, D. D. , and K. K. Kharbanda . 2017. “Dextran Sodium Sulfate Colitis Murine Model: An Indispensable Tool for Advancing Our Understanding of Inflammatory Bowel Diseases Pathogenesis.” World Journal of Gastroenterology 23: 6016–6029. 10.3748/wjg.v23.i33.6016 PMC5597494 28970718

[ptr70404-bib-0010] Fan, T.‐J. , L. Goeser , K. Lu , J. J. Faith , and J. J. Hansen . 2021. “ *Enterococcus faecalis* Glucosamine Metabolism Exacerbates Experimental Colitis.” Cellular and Molecular Gastroenterology and Hepatology 12: 1373–1389. 10.1016/j.jcmgh.2021.06.017 PMC8479252 34246809

[ptr70404-bib-0011] Heinrich, M. , G. Appendino , T. Efferth , et al. 2019. “Best Practice in Research ‐ Overcoming Common Challenges in Phytopharmacological Research.” Journal of Ethnopharmacology 246: 112230. 10.1016/j.jep.2019.112230 31526860

[ptr70404-bib-0012] Hirano, A. , J. Umeno , Y. Okamoto , et al. 2018. “Comparison of the Microbial Community Structure Between Inflamed and Non‐Inflamed Sites in Patients With Ulcerative Colitis.” Journal of Gastroenterology and Hepatology 33: 1590–1597. 10.1111/jgh.14129 29462845

[ptr70404-bib-0013] Huang, Y. , S. Li , Q. Wu , et al. 2023. “Study on the Mechanism of Seven Classic Prescriptions for Treating Ulcerative Colitis Based on Network Pharmacology.” Natural Product Research and Development 35: 662–676.

[ptr70404-bib-0014] Jialing, L. , G. Yangyang , Z. Jing , et al. 2020. “Changes in Serum Inflammatory Cytokine Levels and Intestinal Flora in a Self‐Healing Dextran Sodium Sulfate‐Induced Ulcerative Colitis Murine Model.” Life Sciences 263: 118587. 10.1016/j.lfs.2020.118587 33065145

[ptr70404-bib-0015] Jing, X. P. , X. P. Song , and S. Liu . 2021. “Study on the Therapeeutic Effect and Inflammatory Mechanism of Enema With Modified Baitouweng Decoction in the Treatment of Chronic Ulcerative Colitis.” Shaanxi Journal of Traditional Chinese Medicine 42: 1060–1063.

[ptr70404-bib-0016] Le Berre, C. , S. Honap , and L. Peyrin‐Biroulet . 2023. “Ulcerative Colitis.” Lancet 402: 571–584. 10.1016/S0140-6736(23)00966-2 37573077

[ptr70404-bib-0017] Lee, C. E. , A. Raduka , N. Gao , A. Hussain , and F. Rezaee . 2024. “8‐Bromo‐cAMP Attenuates Human Airway Epithelial Barrier Disruption Caused by Titanium Dioxide Fine and Nanoparticles.” Tissue Barriers 12: 2300579. 10.1080/21688370.2023.2300579 PMC11583697 38166590

[ptr70404-bib-0018] Li, J. , W. X. Zhou , S. Liu , et al. 2019. “Similarities and Differences in Clinical and Pathologic Features of Inflammatory Bowel Disease‐Associated Colorectal Cancer in China and Canada.” Chinese Medical Journal (English) 132: 2664–2669. 10.1097/CM9.0000000000000525 PMC6940096 31725457

[ptr70404-bib-0019] Lou, H. , H. Li , S. Zhang , H. Lu , and Q. Chen . 2021. “A Review on Preparation of Betulinic Acid and Its Biological Activities.” Molecules 26: 5583. 10.3390/molecules26185583 PMC8468263 34577056

[ptr70404-bib-0020] Nadeem, M. S. , V. Kumar , F. A. Al‐Abbasi , M. A. Kamal , and F. Anwar . 2020. “Risk of Colorectal Cancer in Inflammatory Bowel Diseases.” Seminars in Cancer Biology 64: 51–60. 10.1016/j.semcancer.2019.05.001 31112753

[ptr70404-bib-0021] Neurath, M. F. 2019. “Targeting Immune Cell Circuits and Trafficking in Inflammatory Bowel Disease.” Nature Immunology 20: 970–979. 10.1038/s41590-019-0415-0 31235952

[ptr70404-bib-0022] Nishihara, Y. , H. Ogino , M. Tanaka , et al. 2021. “Mucosa‐Associated Gut Microbiota Reflects Clinical Course of Ulcerative Colitis.” Scientific Reports 11: 13743. 10.1038/s41598-021-92870-0 PMC8253849 34215773

[ptr70404-bib-0023] Rochel, N. , J. M. Wurtz , A. Mitschler , B. Klaholz , and D. Moras . 2000. “The Crystal Structure of the Nuclear Receptor for Vitamin D Bound to Its Natural Ligand.” Molecular Cell 5: 173–179. 10.1016/s1097-2765(00)80413-x 10678179

[ptr70404-bib-0024] Sandle, G. I. , and V. M. Rajendran . 2025. “Ion Transport and Epithelial Barrier Dysfunction in Experimental Models of Ulcerative Colitis.” American Journal of Physiology. Gastrointestinal and Liver Physiology 328: G811–G830. 10.1152/ajpgi.00204.2024 PMC12139458 40184259

[ptr70404-bib-0025] Seishima, J. , N. Iida , K. Kitamura , et al. 2019. “Gut‐Derived *Enterococcus faecium* From Ulcerative Colitis Patients Promotes Colitis in a Genetically Susceptible Mouse Host.” Genome Biology 20: 252. 10.1186/s13059-019-1879-9 PMC6876129 31767028

[ptr70404-bib-0026] Tan, Y. , Y. Guan , Y. Sun , and C. Zheng . 2019. “Correlation of Intestinal Mucosal Healing and Tight Junction Protein Expression in Ulcerative Colitis Patients.” American Journal of the Medical Sciences 357: 195–204. 10.1016/j.amjms.2018.11.011 30638599

[ptr70404-bib-0027] Triantos, C. , I. Aggeletopoulou , G. J. Mantzaris , and A. Mouzaki . 2022. “Molecular Basis of Vitamin D Action in Inflammatory Bowel Disease.” Autoimmunity Reviews 21: 103136. 10.1016/j.autrev.2022.103136 35792343

[ptr70404-bib-0028] Walker, A. W. , J. D. Sanderson , C. Churcher , et al. 2011. “High‐Throughput Clone Library Analysis of the Mucosa‐Associated Microbiota Reveals Dysbiosis and Differences Between Inflamed and Non‐Inflamed Regions of the Intestine in Inflammatory Bowel Disease.” BMC Microbiology 11: 7. 10.1186/1471-2180-11-7 PMC3032643 21219646

[ptr70404-bib-0029] Wang, W. , Z. Liu , W. Yue , et al. 2022. “Mucosa‐Colonizing Microbiota Correlate With Host Autophagy Signaling in Patients With Inflammatory Bowel Disease.” Frontiers in Microbiology 13: 875238. 10.3389/fmicb.2022.875238 PMC9178242 35694307

[ptr70404-bib-0030] Wang, X. , A. A. Izzo , A. Papapetropoulos , et al. 2024. “Natural Product Pharmacology: The British Journal of Pharmacology Perspective.” British Journal of Pharmacology 181: 3547–3555. 10.1111/bph.17300 39128855

[ptr70404-bib-0031] Wu, M. , P. Li , J. Li , Y. An , M. Wang , and G. Zhong . 2020. “The Differences Between Luminal Microbiota and Mucosal Microbiota in Mice.” Journal of Microbiology and Biotechnology 30: 287–295. 10.4014/jmb.1908.08037 PMC9728297 31635444

[ptr70404-bib-0032] Yan, Z.‐X. , Y.‐M. Liu , T. Ma , et al. 2020. “Efficacy and Safety of Retention Enema With Traditional Chinese Medicine for Ulcerative Colitis: A Meta‐Analysis of Randomized Controlled Trials.” Complementary Therapies in Clinical Practice 42: 101278. 10.1016/j.ctcp.2020.101278 33276227

