# Supplementary material for: Betulinic Acid Enema Alleviates Colitis by Targeting Vitamin D Receptor to Protect the Intestinal Mucosal Barrier and Modulate Mucosa‐Associated Microbiota
Source: Phytother Res. 2026 Jun 18;40(11):6094–111. doi: 10.1002/ptr.70404 (PMC13753679; doi:10.1002/ptr.70404)
Supplement: Supplementary file 1 [file PTR-40-6094-s001.docx]

| Supplied table1: Primers sequence information for qPCR | | |
| --- | --- | --- |
| *Gene* | Forward primer | Reverse primer |
| *β-actin* | 5′-GTCAGGTCATCACTATCGGCAAT-3′ | 5′-AGAGGTCTTTACGGATGTCAACGT-3′ |
| *TNFα* | 5′-CCCCAAAGGGATGAGAAGTTC-3′ | 5′-CCTCCACTTGGTGGTTTGCT-3′ |
| *IL6* | 5′-CCAGAAACCGCTATGAAGTTCC-3′ | 5′-GTTGGGAGTGGTATCCTCTGTGA-3′ |
| *IL1β* | 5′-GTTCCCATTAGACAACTGCACTACAG-3′ | 5′-GTCGTTGCTTGGTTCTCCTTGTA-3′ |
| *iNOS* | 5′‐GAACTGTAGCACAGCACAGGAAAT-3′ | 5′‐CGTACCGGATGAGCTGTGAAT-3′ |
| *COX2* | 5′‐CAGTTTATGTTGTCTGTCCAGAGTTTC-3′ | 5′‐CCAGCACTTCACCCATCAGTT-3′ |
|  | | |
